# The impact of housing prices on residents’ health: a systematic review

**DOI:** 10.1186/s12889-024-18360-w

**Published:** 2024-04-01

**Authors:** Ashmita Grewal, Kirk J. Hepburn, Scott A. Lear, Marina Adshade, Kiffer G. Card

**Affiliations:** 1 Faculty of Health Sciences, Simon Fraser University, Blusson Hall, 8888 University Dr. , Burnaby, BC V5A 1S6 Canada; 2 Vancouver School of Economics, University of British Columbia, Vancouver, BC Canada

**Keywords:** Health, Well-being, Housing price, Economic stress, Social determinants of health

## Abstract

**Background:**

Rising housing prices are becoming a top public health priority and are an emerging concern for policy makers and community leaders. This report reviews and synthesizes evidence examining the association between changes in housing price and health outcomes.

**Methods:**

We conducted a systematic literature review by searching the SCOPUS and PubMed databases for keywords related to housing price and health. Articles were screened by two reviewers for eligibility, which restricted inclusion to original research articles measuring changes in housing prices and health outcomes, published prior to June 31st, 2022.

**Results:**

Among 23 eligible studies, we found that changes in housing prices were heterogeneously associated with physical and mental health outcomes, with multiple mechanisms contributing to both positive and negative health outcomes. Income-level and home-ownership status were identified as key moderators, with lower-income individuals and renters experience negative health consequences from rising housing prices. This may have resulted from increased stress and financial strain among these groups. Meanwhile, the economic benefits of rising housing prices were seen to support health for higher-income individuals and homeowners – potentially due to increased wealth or perception of wealth.

**Conclusions:**

Based on the associations identified in this review, it appears that potential gains to health associated with rising housing prices are inequitably distributed. Housing policies should consider the health inequities born by renters and low-income individuals. Further research should explore mechanisms and interventions to reduce uneven economic impacts on health.

**Supplementary Information:**

The online version contains supplementary material available at 10.1186/s12889-024-18360-w.

## Introduction

In contemporary society, the structures we live in, as well as our legal relationships to these structures, are intertwined with our fundamental senses of self and belonging [1–3]. For decades, homeownership has been recognized as a core measure of success [4, 5]. Recognizing the importance of housing, studies have variously examined the effects of wide-ranging housing-related factors on health, including housing quality, overcrowding, neighbourhood deprivation, social cohesion, housing density, housing suitability or sufficiency, and neighbourhood socioeconomic status [6, 7]. While these effects continue to be explored, it is generally agreed that housing is a fundamental determinant of health [7], which broadly exerts impacts on health through a variety of mechanisms.

Indeed, housing-related health effects arise from specific housing conditions, as well as the legal conditions that define our relationships to these spaces, and our emotional attachments to these various factors. For example, living and owning a home can create access to opportunities that can further bolster health [8]. Similarly, housing related factors—such as indebtedness, mortgage stress, and credit problems—can cause severe mental health problems, depression, and suicide ideation [9, 10]. With these factors in mind, people in most countries face numerous barriers to securing their right to a home [5, 11], and a wide array of policies have been proposed and implemented to address these barriers [12–14]. In addition to these factors, the location of a home, the quality of a building, or the neighbourhood context in which a home exists are also hugely influential to health [7, 15, 16].

In conceptualizing these varied mechanisms, it is important to consider both direct and indirect mechanisms through which the relationship between housing and health manifests. Direct effects predominantly emerge from psycho-physiological stress responses. Elevated housing costs can induce chronic stress, leading to mental health conditions, like anxiety and depression, and other health problems [17]. Indirectly, escalating housing prices exert economic pressures that limit individuals' capacity to allocate resources towards health-promoting activities and necessities. This economic strain can result in compromised nutrition, reduced access to healthcare services, and diminished ability to manage chronic conditions, therefore, exacerbating health disparities. Moreover, the financial burden can lead to other lifestyle changes that further impair physical and mental well-being, such as increased substance use or reduced physical activity.

Despite these effects being documented in previous studies, there are no systematic reviews on the impact of rising housing prices on health. The present review aims to examine the effect of housing price on health by considering whether changes in housing market price impact the health of residents living in an area. To accomplish this aim, we conduct a systematic review. This review is especially timely since housing prices have risen in the past five years at an alarming rate.

## Methods

### Article search

The first step in our multi-stage systematic literature review was to manually identify relevant articles through a rudimentary search on SCOPUS and PubMed (Appendix B). We then created a list of keywords to use for our search. Keywords aimed to identify articles that measured changes in housing prices and health impacts, Appendix A outlines how we identified keywords and provides a complete list of selected keywords. After conducting the keyword search in PubMed and SCOPUS, duplicates were removed and the remaining articles were then uploaded to Rayyan, an online software that aids in systematic reviews [18]. To assess whether our search is comprehensive, AG confirmed that the articles identified in the rudimentary initial search, mentioned earlier, were also included in this search. For the purposes of this literature review, we define health using the language provided by the World Health Organization (1948): “health is a complete state of mental, physical, and social well-being, and not merely the absence of disease.” As such, no additional inclusion or exclusion criteria were used to exclude or include specific health conditions. We felt this was appropriate given that this is the first literature review on this topic and because after a review of included articles, it was apparent that a wide variety of health outcomes have been considered. Furthermore, the biopsychosocial models of health that we engage to inform our view that housing prices have direct and indirect effects on health underscore that diverse and nuanced pathways across various mental and physical domains of health are likely important to consider. Using Rayyan, AG and LW reviewed the titles of each manuscript to remove articles that were clearly not relevant to this review [18]. The application of inclusion and exclusion criteria resulted in 21 articles that were directly relevant to this review. AG and LW also searched the reference lists for these 21 articles to identify any additional articles. These missed articles were added to our final inclusion list, creating a total of 23 included articles.

### Data extraction

Data were extracted by AG and LW from each of the identified and included articles and AG re-reviewed the data extraction to verify accuracy. Extracted variables included: first author name, year of publication, years of data collection, sample size, location(s) of study, study design (e.g., case control, cohort, cross-sectional, serial cross-sectional study), analysis type (e.g., regression), outcome, explanatory factor, confounders/mediators/moderators, and a summary of primary findings (including effect size measures). This data extraction is provided as Table 1.

**Table 1:** Data Extraction for Included Studies

Article	Study Design	Variables	Primary Findings
**Yue & Ponce (2021)** [19] **N:** 34,182 people **Location:** United States **Timeframe:** 1996–2016	**Data:** Longitudinal study with secondary analyses of Health and Retirement Study with linkage to zip-code level housing price data using Federal Housing Finance Agency **Analyses:** Fixed-effects panel regression model with interaction term to test effect modification by housing status Results reported in relative ratio	**Outcome(s):** Self-reported health (excellent/very good/good vs. fair/poor); Centre for Epidemiological Studies Depression Scale (CESD-8, 0–8 scores); Obesity (Body Mass Index ≥ 30 vs < 30); Current smoking (yes vs. no) **Explanatory Factor(s):** Housing Price Index scores (i.e., zip-code level housing price index scores from the U.S. Federal Housing Finance Agency [FHFA]) **Moderator(s)/Mediator(s):** Housing status (outright owner, mortgaged owner, renter) **Confounder(s):** Age, Gender, Birthplace, Race/Ethnicity, Educational attainment, Marital status, Employment status, County-level poverty rate, County-level median income, County-level unemployment, County-level number of hospital beds	- A 100% in HPI (Housing Price Index) scores is associated with 3.5% higher likelihood of reporting excellent/very good/good health status for renters, and 2.8% higher likelihood for mortgaged owners, both *P* < 0.01). For outright owners a 100% increase in HPI is associated with a 2.1% increase in reporting excellent/very good/good health though the association is not statistically significant (*P *< 0.1) - For outright owners a 100% increase housing prices leads to a 2.7% decrease in CES-D scores, and a 3.96% decrease for mortgaged owners, though both are not statistically significant. For renters, a 100% increase in HPI leads to a 23.69% decrease in CES-D scores, (*P* < 0.01) - A 100% increase in HPI leads to a 1.82% decrease in obesity for outright owners (*P* < 0.05) and 2.85% decrease for renters (*P* < 0.01). 100% increase in HPI leads to a 0.38% decrease in obesity for mortgaged owners, not statistically significant - For outright owners, a 100% increase in HPI leads to a 0.95% increase in smoking, and for mortgaged owners a 0.03% decrease in smoking, both statistically insignificant. For renters, a 100% increase in HPI leads to a 3.03% decrease in smoking, *P* < 0.01)
**Chen et al. (2021**) [20] **N:** 8318 people **Location**: China **Timeframe:** 2015	**Data:** Cross Sectional study design involving the Chinese General Social Survey and using Real Estate Statistics Database to obtain data on average selling price **Analysis**: Multivariate ordered logistic regression models, results reported as coefficients	**Outcome:** Self rated physical health (what do you think of your current state of physical health – rate 1–5, very unhealthy – very healthy); Self rated mental health (how often have you felt depressed or depressed in the last four weeks – rate 1–5, always – never) **Explanatory**: Average selling price from the Real Estate Statistics Database **Mediating/Moderator:** Homeowner vs. renter; Related affordability; Reduction in purchasing power **Confounding:** Age; Gender; Marriage; Political status; Income; Work status; Household residence type	- Housing prices were negatively related to self-rated health for east (-0,573, *P* < 0.01) and central residents (-0.707, *p *< 0.05), but not statistically significant for west residents (-0.637, *P* < 0.559) -Housing prices are positively related to mental health among eastern respondents (0.332, *p* < 0.01), but a negative impact on central residents’ mental health (-0.040, *P* = 0.912), and negative impact on west respondents’ mental health (-0.457, *P* = .417)
**Arcaya et al. (2020) **[21] **N:** 300 municipalities **Location:** municipalities in Boston metro area **Timeframe**: 2015–2020	**Data:** Cross Sectional study design. The qualitative component of this study includes 40–80 min in depth, semi-structured interviews. The quantitative data includes COVID19 case numbers from the Massachusetts Department of Public Health. Municipal level housing prices were created using Zillow data **Analysis:** Semi-structured participant interviews with content analysis & ordinary least squares regression models using COVID-19 rate per 100,000	**Outcome:** Increased/decreased incidence of COVID19 cases (COVID19 data obtained from Massachusetts department of public health) **Explanatory:** Five-year change in municipal level housing values (Zillow Home Value Index of 2015 and 2020) burden of housing cost for low-income households **Confounding**: Share of population foreign born, living in poverty, proximity to Boston, crowding and density, racial/ethnic composition, work from home, and other suspected community-level differences **Mediator/Moderating:** Crowding; Doubling up; Homelessness; Part-time work	- An increase of 1% in housing value, since 2015, is associated with an increase in 14 additional cases of COVID19 per 100,000. (*P* < 0.05)
**Lee et al. (2021) **[22] **N:** 2556 quarterly observations of Taiwan housing index **Location:**China **Timeframe:** 2001–2011	**Data:** Longitudinal study with secondary analysis of National Health Insurance Research Database (NHIRD) & the Taiwan Housing Index (TH) **Analysis:** Distributed lag nonlinear model (DLNM), results reported as relative risks	**Outcome**: Increase/decrease in antidepressant prescription incidence using ambulatory care data and inpatient expenditure data from the longitudinal health insurance database **Explanatory:** Fluctuations in housing market using the Taiwan housing index **Moderating/Mediating**: Socioeconomic status; Homeowner vs. buyer **Confounding**: SARS, financial crises; Stock; Long-term trends	- A 13.3% increase in antidepressant prescriptions was observed when housing index peaked at 170.13 (*p* < 0.05)
**Fichera & Gathergood (2016) **[23] **N:** 105,170 individual person-years of observation **Location**: United Kingdom **Timeframe:** 1993–2008	**Data:** A longitudinal study using the British Household Panel Survey **Analysis:** Various fixed effect models, and results reported as regression coefficients	**Outcome:** Number of health conditions (13 asked); Self-assessed health (1–5, excellent – very poor); Depression; GHQ-12 for psychological health (0-poorest, 12-highest) **Explanatory:** Housing price data **Confounding:** Country level employment, annual income; Work-related behaviors **Mediating/Moderating:** Labor market activity (Hours of work); Health care coverage	-A 100% increase in housing prices, leads to a 0.0819 decrease in multiple health conditions (*p* < 0.01) -A 100% increase housing prices leads to a 0.0377-point decrease in self assessed health (*P* < 0.01) -A 100% increase in housing prices leads to a 0.00491-point decrease in depression (statistically insignificant) -A 100% increase in housing prices leads to a 0.0313-point decrease in general health questionnaire (statistically insignificant)
**Kim et al. (2021) **[24] **N:** 423 "units of analysis" or neighborhoods **Location**: South Korea **Timeframe**: 2013–2018	**Data:** Cross Sectional study using data from the Ministry of Interior and Safety. Neighborhood housing price data obtained from the Ministry of Land, Infrastructure, and Transport. Supplementary data obtained from the Seoul Metropolitan Government’s information disclosure system **Analysis**: Mapping distribution of all-cause mortality and housing prices using pooled OLS models	**Outcome:** All-cause mortality using Ministry of Interior and Safety data **Explanatory:** Median houses price using the Ministry of Land, infrastructure, and transport data in Korea **Confounding**: Poverty rate; Population density; Business workers; Number of nearby subway stations; Movement to other neighborhoods; Social and Public Health Policies **Mediating/Moderating:** Education; Access to resources; Socioeconomic development of area	-A 1% increase in housing prices was related to a 0.05% decrease in all-cause mortality
**Hamoudi & Dowd (2014) **[25] **N:** 4207 people **Location:** United States **Timeframe:** mid 1990s to mid 2000s	**Data:** Longitudinal study using data from the Health and Retirement Study (HRS), and housing data from DataQuick **Analysis**: Regression modeling, results reported as regression coefficients	**Outcome**: Depression measured with CES-D; Beck Anxiety inventory; Mroczeck/Korarz Positive Affect Inventory; Mrocczek/Kolarz Negative Affect Inventory **Explanatory:** Housing values from DataQuick **Confounding:** Home value at baseline; Total non-housing wealth; Share of housing equity at baseline; Birth year; Sex; Area of residence; Self-rated health; Indicators for smoking and exercise at baseline; Share of housing equity at baseline; Study cohort **Mediating/Moderating:** Homeowner vs. renter; Wealth; Local area improvements	- Movement from 10th-90th percentile for homeowners in terms of housing appreciation is associated with: - decreased likelihood of anxiety in homeowner females (-19.6), and increased likelihood of anxiety in male homeowners (1.7) - (b) decreased depression risk among female homeowners (-2.3), and decreased depression among male homeowners (-1.3) - decrease likelihood of negative effect among female homeowners (-3.5) and decrease likelihood in negative effect for male homeowners (-7.7) - increase in likelihood of positive affect for female homeowners (5.9), and male homeowners (1.9) - increase in likelihood of anxiety for female renters (2.9), and increase in likelihood for male renters (68.2) - increase in likelihood of depression for female renters (15.7), and increase in likelihood for male renters (16.8) - increase in likelihood of negative effect for female renters (27.7) and increase for male renters (35.8) - decrease in likelihood for positive affect among female renters (-18.7) and increase for male renters (8.3)
**Yuan et al. (2020**) [26] **N:** 34,000 people **Location:** China (25 provinces) **Timeframe:** 2010 and 2014	**Data:** Cross sectional study using data from the 2010 and 2014 China Family Panel Study **Analysis:** Regression analysis, reported as coefficients	**Outcome:** Physical health—"What do you think of your health" (quite healthy, very healthy, healthy = 1, normal, unhealthy-0); Mental acuity—"Can you remember the main things that happened to you in a recent week"(remember all, remember most = 1, remember half, remember a few, remember a little = 0); Emotional well-being—"How often have you felt upset, depressed, and unable to do anything in the last month", and "How often have you felt nervous in the last month (1–5 – almost every day – never, the average of two answers taken for emotional well-being score)" **Explanatory:** Average annual growth rate of residential housing price over past five years **Confounding:** Gender; Age; Years of education; Smoking; Exercise; Income; Housing ownership; City-level factors **Mediating/Moderating:** Social status seeking behavior	-Rising housing prices negatively impacts the physical health (-0.575, *P* < 0.01), mental acuity (-0.198, *P* < 0.01), and emotional well-being (-0.092, *P* < 0.01) of middle-aged and elderly people
**Atalay et al. (2017)** [27] **N**: 19,000 people **Location:** Australia **Timeframe:** 2001–2015	**Data:** Serial cross-sectional using data from the RP Data Historical house price dataset, and the Household, Income, and Labor Dynamic in Australia Survey (HILDA) **Analysis:** Fixed effects model w/multiple linear regression analysis	**Outcomes**: Mental and physical health measures from the 36-item short form health survey (average of four responses taken to calculate a single score for mental health and physical health scores) **Explanatory:** Local area median house price series from RP Data Historical data set **Confounding:** Demographic controls; Employment; Year fixed effects; Individual LGA fixed effects; LGA level employment; LGA level average income; Household income; Tenure status (mortgaged owner, outright owner, renter) **Mediating/Moderating**; N/A	-A one standard deviation increase in house prices leads to an increase in physical health for outright owners (*p* < 0.05), but the impact is statistically insignificant on the mental health for outright owners -For renters, a one standard deviation increase in house prices leads to a decrease in physical health (*p* < 0.01), and a 0.801 decrease in mental health (*p* < 0.01) -For mortgaged owners, the impact of house price growth on the physical and mental health is statistically insignificant
**Bao et al. (2022)** [28] **N:** 9 countries **Location:** Canada, France, Japan, Netherlands, Spain, Switzerland, Sweden, United Kingdom, USA **Timeframe**: 1996–2019	**Data:** Cross Sectional Study design, using data for housing rent from the OECD, and the World Bank for the remaining variables **Analysis:** The study adopts the fixed effect model (FEM) and random effect model (REM) methods, reporting as regression coefficients	**Outcomes:** Infant mortality rate (per 1,000 live births); Life expectancy at birth in total years **Explanatory:** Housing rent prices are taken at the base of 2015. Data for house rent is extracted from the OECD **Confounding**: GDP per capita; Health expenditures; Unemployment **Mediating/Moderating**: Real estate development (including health facilities); Consumption	-In Fixed Effect Model (FEM) 1% increase in house rent leads a reduction in infant mortality rate (-0.020, *p* < 0.05) and increases life expectancy (0.040, *p* < 0.1) -In the Random Effect Model (REM), a 1% increase in house rents leads to decrease in infant mortality rate, (-0.027, *P* < 0.01) and an increase in life expectancy (0.089, *P* < 0.01)
**Daysal et al. (2021)** [29] **N:** 204,507 people **Location:** Denmark **Timeframe:** 1992–2011	**Data;** Longitudinal study; Birth registry, including hospital or home births and infant health. National patient registry provides information for hospital visits. Housing data comes from the States Scales and Valuation Registry **Analysis:** Secondary analysis	**Outcomes:** Birth weight; Pre-maturity – birth Registry; Number of days hospitalized; Number of emergency room visits – national Patient Registry **Explanatory:** Price changes for homeowners and non-homeowners from the States Scale and Valuation Registry in Denmark **Confounding:** Household income; Years of education; Number of children; Partner; Unemployed; Age **Mediating/Moderating**: Health investments; Income effect	- 100,000 DKK (Danish Krone) increase in house prices leads to a 0.15% decrease in the likelihood of being premature (*p* < 0.05), and a 0.05 percentage point reduction in the likelihood of low-birth weight (statistically insignificant)
**De & Segura-Escano (2021)** [30] **N:** 3.1 million people **Location:** United States **Timeframe**: 2005–2012	**Data:** Cross sectional method using the Behavioral Risk Factor Surveillance System in relation to home values per square foot, assessed using the Zillow Home Value Index **Analysis:** Several variations of a linear probability model, reported as beta coefficients	**Outcome**: Current drinker (assigned 1 if drank in the past 30 days); Binge drinker (assigned 1 if drank 5 drinks or more for female, four or more drinks for males on one occasion); Excessive drinker (1 = more than 30 drinks a month for women, 1 = more than 30 drinks per month for men); Alcohol intensity (number of drinks consumed in one day, and number of days consumed alcohol in the past 30 days **Explanatory:** Zillow home price index **Confounding/control variables**: Gender; Marital status; Race/ethnicity; Education; Employment status; Income; Country-level characteristics (taxes on beer/alcohol, smoking laws, population density, country median income, country college ratio, country diversity); Homeownership (owner vs. renter) **Mediating/moderating:** Stress/mental health	1% decrease in Zillow Home Value Index for homeowners - leads to an increase in being a current drinker (beta = 0.0013, *P* < 0.01) - leads to an increase in being a binge drinker (beta = 0.0003, *P* < 0.01) - leads to an increase in being an excessive drinker (beta = 0.0002, *P* < 0.01) - leads to an increase in drinks per day (beta = 0.0007, *P* < 0.01), and days of alcohol in past 30 days (beta = 0.0169, *P* < 0.01) 1% decrease in Zillow Home Value Index for renters - leads to an increase in being a current drinker (beta = 0.0003, *P* < 0.10) - leads to a decrease in binge drinker (beta = -0.0001, statistically insignificant) - leads to an increase in excessive drinking (beta = 0.0003, statistically insignificant) - leads to an increase in drinks per day (0.0004, statistically insignificant), and increase in days of alcohol in past 30 days (0.0056, *P* < 0.01)
**Wang & Liang (2021)** [31] **N:** 51, 258 people **Location**: China **Timeframe:** 2014, 2016, 2018	**Data:** Cross sectional including data the China Family Panel Studies in 2014, 2016, 2018, and the housing price data is from the China Statistical Yearbook in 2014 and 2018 **Analysis**: Econometric regression model	**Outcome:** CES-D scores to assess psychological health; Five questions assess physical health: Self-rated physical health (How do you think your health status is?); Recent changes in health status (scale 1–3, 1-Worse, 2-No change, 3-Better); Recent physical discomfort (Binary, Yes/No); Degree of physical illness and injury, and chronic disease"(1–3, 0-No disease or injury 2-Moderate, 3- Serious); Chronic disease (Binary, Yes/No) **Explanatory:** House price data from the China Family Panel Study by the Chinese Social Science Survey **Confounding/control variables:** Personal; Family; Regional characteristics; Sex; Age; Education; Employment status; Weekly exercise duration; Homeownership (Owner vs. without houses); Housing area size; Total family property; Net assets; Disposable income; Number of urban health technicians **Mediating/Moderating**: Health behaviors; Area level improvements	For residents who own houses, with mortgage, 10% increase in housing prices - leads to decrease in CES-D scores (-0.103, *P *< 0.01) - leads to decrease in self-assessment of physical health status (-0.038, *P* < 0.05) - leads to a greater likelihood of reporting positive changes in health status (0.019, statistically insignificant) - leads to greater likelihood of decrease in degree of physical disease and injury (-0.042, statistically insignificant) - decrease in physical discomfort (-0.046, *P* < 0.01) - leads to increase in prevalence of chronic disease (0.006, statistically insignificant) For residents who own homes without mortgages, a 10% increase in housing prices leads to - increase in CES-D scores (0.072, *P* < 0.05) - increase in reporting better physical health status (0.026, *P* < 0.01) - decrease in reporting positive changes in health status (-0.009, *P* < 0.10) - decrease in likelihood of reporting physical discomfort (-0.025, *P* < 0.01) - decrease reporting physical disease or injury (-0.028, *P *< 0.01) - decrease in probability of chronic disease (-0.001, *P* < 0.01) For residents without houses, with mortgages (previous loan), a 10% increase in housing prices leads to - decrease in CES-D scores (-0.091, statistically insignificant) - decrease in self-assessment of physical health (-1.10, *P* < .10) - increase in better changes in health (1.41, *P* < 0.05) - decrease in recent physical discomfort (-1.71, *P* < 0.01) - decrease in degree of physical disease and injury (-1.5, *P* < 0.01) - decrease in reporting chronic disease (-0.025, statistically insignificant) For residents without houses, without mortgages 10% increase in housing prices leads to change in probability of reporting: - increase in CES-D scores (0.024, *P* < 0.10 - decrease in physical health status (-0.0005, statistically insignificant) - increase in better changes in health (0.023, *P* < .10) - decrease in physical discomfort (-0.065, *P* < 0.01) - decrease in degree of physical injury and illness (-0.07, statistically insignificant) - decrease in reporting chronic disease (-0.025, *P* < 0.01)
**Wei et al. (2021)** [32] **N:** 1116 observations from 32 cities Each observation contains information on mental health and the monthly house price growth rate of each city **Location**: China **Timeframe:** 2013–2017	**Data:** Longitudinal study with secondary analysis of serial cross-sectional (monthly) Chinese data using the China Health Insurance Research Association claim database; quality adjusted house price index **Analysis:** Fixed-effect models	**Outcome:** Mental health consultation was measured using the China Health Insurance Research Association claims database, which included the rate at which the people consulted physicians regarding mental health concerns **Explanatory:** Quality adjusted house price index- growth rate was computed at 1, 3, 6, 12 months **Confounding/Control:** Disposable income; City fixed effects; Year by month fixed effects **Mediating/Moderating:** Housing affordability (percentage of income spent on housing); Marriage prospects within Chinese culture favors males or males’ family who can afford to buy a home	-Increase of one standard deviation in the past 3 months leads to an increase of 0.0443 standard deviation in consultation rate for mental disorders. (*p* < 0.05) -House price growth rates in the past 1, 3, and 6 months has a statistically significant impact on the consultation rate, however, not for house price growth in the past 12 months, suggesting a short-term impact rather than a long-term impact
**Zhang & Zhang (2019)** [33] **N:** 9414 people **Location:** 28 provinces of Mainland China **Timeframe:** 2011	**Data:** Cross sectional secondary analysis of China Household Finance Survey **Analysis:** Ordered prohibit model, coefficients reported	**Outcome:** Subjective well-being (5-point Likert scale, 1- very unhappy, 2- unhappy, 3- neutral, 4- happy, 5-very happy) **Explanatory:** 2011 China Household Finance Survey—house value appreciation; Home ownership; House value at the time of purchase; Current house value **Mediating/Moderating:** Homeownership; Income; Region of residence **Confounding:** not listed	-A 1% increase in home values leads to an increase in the subjective well-being of homeowners. (0.070, *p* < 0.01)
**Feng & Nie. (2022)** [34] **N:** 44,495 observations physical health. 29,647 observations mental health **Location:** China **Timeframe:** 2012, 2014, 2016	**Data:** Cross- sectional secondary analysis of China Family Panel Study; House price data came from the *CEInet* statistics database **Analysis:** Regression modelling, coefficients reported	**Outcome:** Physical health measured using four health indicators leading to a complex index (ranging from 0–1); Mental health measured by average value from 20 questions (ranging from 1 to 4). Higher the value, worse the mental and physical health **Explanatory:** Housing price data from *CEInet* statistics database **Mediating/Moderating**: Number of owner-occupied houses; Net housing value **Confounding:** Year fixed effects; Regional year fixed effects; Country-individual fixed effects	-Housing prices have a significant positive impact of the physical healthof residents (-0.050, *P* < 0.05) and a negative impact on mental health (0.161, *P* < 0.01)
**Chun (2020)** [35] **N:**191,121 people **Location:** South Korea **Timeframe:** 2009–2015	**Data:** Longitudinal design using Korea Health Panel data; Korea Appraisal Board data for the housing price index **Analysis:** Empirical analysis of the impact of house price changes on depression, coefficients, and OR reported	**Outcome:** Depression (as a proxy of mental health) **Explanatory:** Korea appraisal board housing price data **Confounding:** Sex; Age; Marital status; Number of households members; Education level; Status of employment; Income; Smoking; Drinking **Mediating/Moderating;** Homeownership vs. renters,	-The rise in housing prices, decreases likelihood of depression for homeowners (-0.01, *P* < 0.05), and renters (-0.01, statistically insignificant)
**Xu & Wang (2021)** [36] **N:** 9,515 people **Location:** 9 provinces of China **Timeframe**: 2000–2011	**Data:** Longitudinal study design using China Health and Nutrition Survey (CHNS) including data on health status and behaviors. Housing price data from the China Real Estate Statistics Yearbook. The sample includes working age individuals between the age of 15–60 **Analysis:** Use an instrumental variable approach, coefficients reported	**Outcome:** Incidence of chronic diseases **Explanatory:** Province level data from the China Real Estate Statistics Yearbook **Mediating/Moderating:** Culture; Marriage prospects **Control variables:** Number of medical personnel per 1,000 residents; Wastewater emissions and sulfur dioxide emissions; GDP per capita; Regional level fixed effects; Individual level fixed effects	-Result show that a 10% increase in housing prices leads to an increase the prevalence of chronic diseases (0.329, *P* < 0.01)
**Hamoudi, & Dowd (2013) **[37] **N:** 4207 people **Location:** United States **Timeframe:** 1992–2006	**Data:** Quasi experimental design; Secondary analysis of Health and Retirement study and housing data from Data Quick **Analysis:** Regression modelling, coefficients reported	**Outcome:** Capacity for daily living activities; Incidence of cardiovascular disease (Yes or No); Peak expiratory flow—measured with the Mini Wright Peak Flow Meter; Balance test; Timed walk task for participants aged 65 + ; Waist circumference; Diastolic and systolic blood pressure **Explanatory:** Natural logarithm of home values in 2006 **Confounding**: Home value at baseline; Total non-housing wealth; Share of housing equity at baseline; Non-housing debt at baseline; Indicators for self-rated health at baseline; Area of residence; Birth year; Race; Gender **Mediating/Moderating:** Homeownership vs. renters; Percent of wealth in home; Health investments	- An increase in housing prices was related to, For all homeowners, - a decrease in ADL difficulties (-6.3, *P* < 0.05) - increase in full balance (10.4, *P* < 0.01) -decrease in timed walking (-0.07, *P* < 0.66) -decrease in lung capacity L/min (-0.75, *P* < 0.94) - decrease in waist circumference (-1.2, *P* < 0.06) - decrease in incident CVD (-1.0, *P* < 0.73) -increase in systolic blood pressure (1.8, *P* < 0.42) -increase in diastolic blood pressure (0.55, *P* < 0.67) For renters, an increase in housing prices was related to: - increase in ADL difficulties (5.6, *P* < 0.38) - decrease in full balance (-3.6, *P* < 0.73) - increase in times walking (0.22, *P* < 0.71) - decrease in lung capacity (-45.7, *P* < 0.08) - increase in waist circumference (1.3, *P* < 0.36) - increase in incident CVD (3.7, *P* < 0.58) - decrease in systolic blood pressure (-7.4, *P* < 0.38) - decrease in diastolic blood pressure (-3.3, *P* < 0.3
**Sung & Qiu. (2020)** [38] **N:** 983, 277 people **Location:** United States **Timeframe:** 2002–2012	**Data:** Serial cross-sectional study and data is from Behavioral Risk Factor Surveillance System, multiple surveys comprised into a single constructed dataset. The house price changes data comes from the MSA Freddie Mac House Price Index and the MSA median rent levels **Analysis:** Fixed effect and time-series regression modeling, coefficients reported	**Outcome:** Self-assessed health is reported as a five-level ordinal variable (excellent – poor); Physical and mental health reported as count variables (number of physical/mentally unhealthy days in past 30 days); Obesity, Exercise, Smoking, Binge drinking, Flu shot, Seatbelt usage– yes/no, dichotomous variables; BMI, Average drinks per day, Binge drinking—continuous variables **Explanatory:** Monthly MSA Freddie Mac House Price Index **Confounding:** Household income; Education level; Age; Race; Marital status **Mediating/Moderating**: Homeowners vs. tenants **Confounding:** Rent levels; Economic conditions; Unemployment rate; Health infrastructure	-An increase of one standard deviation in the Freddie Mac Housing Price Index, leads to For homeowners: - decrease in reporting excellent health (-0.0021, statistically insignificant) - decrease in reporting very good health (-0.0007, statistically insignificant) - increase in reporting fair health (0.0010, statistically insignificant) - increase in reporting poor health (0.0004, statistically insignificant) - decrease in physically bad days (-0.0449, statistically insignificant) - increase in mentally bad days (0.1001, *P* < 0.05) - increase in any exercise (0.0020, statistically insignificant) - increase in moderate exercise (0.0008, statistically insignificant) - increase in vigorous exercise (0.0070, statistically insignificant) - increase in being a current smoker (0.0054, *P* < 0.01) - decrease in smoking everyday (-0.0032, statistically insignificant) - increase in average drinks (0.0424, statistically insignificant) - increase in binge drinking (0.0312, *P* < 0.10) - decrease in flu shot (-0.0042, statistically insignificant) - decrease in wearing seatbelt (-0.0018, statistically insignificant) - increase in drunken driving (0.0131, statistically insignificant) For renters: - decrease in reporting excellent health (-0.0112, *P* < 0.01) - decrease in reporting very good health (-0.0069, *P* < 0.01) - increase in reporting good health (0.0064, *P* < 0.01) - increase in reporting fair health (0.0087, *P* < 0.01) - increase in reporting poor health (0.0030, *P* < 0.01) - increase in physically bad days (0.0998, statistically insignificant) - increase in mentally bad days (0.378, *P* < 0.01) - decrease in BMI (-0.1205, statistically insignificant) - increase in obesity (0.0021, statistically insignificant) - increase in any exercise (0.0020, statistically insignificant) - increase in moderate exercise (0.0008, statistically insignificant) - increase in vigorous exercise (0.0045, statistically insignificant) - increase in being a current smoker (0.0084, statistically insignificant) - decrease in smoking everyday (-0.0037, statistically insignificant) - increase average drinks (0.252, *P* < 0.01) - increase in binge drinking (0.0574, statistically insignificant) - decrease in flu shot (-0.0057, statistically insignificant) - decrease in wearing a seatbelt (-0.0070, statistically insignificant) - decrease in drunken driving (-0.0099, statistically insignificant)
**Wong et al. (2020)** [39] **N:** 163,651 people **Location**: United States **Timeframe:** 2011–2015	**Data:** Cross-sectional observational study using data from the Behavioral risk factor surveillance system and the metropolitan statistical area home and rental value prices from Zillow **Analysis:** Regression modelling, average marginal effects reported	**Outcome:** 6-item brief dietary assessment—Dark green vegetables; Orange vegetables; Other vegetables; Legumes; Whole fruit; 100% fruit juice. Respondents asked number of times food eaten and if eaten at least twice per week **Explanatory:** Housing value measured using the Zillow Home Value Index **Mediating/Moderating**: Homeowner vs. renters; Educational attainment; Race/ethnicity **Confounding/Controlling-** Race/ethnicity; Education; MMSA level aggregate food price; Age; Educational attainment; Marital status; Income categories	-When MMSA-level home rental prices increase by $100: - decrease in the frequency of eating vegetables per week (-0.028, statistically insignificant) - decrease in the frequency of eating fruits per week (-0.108, statistically insignificant) - decrease in the frequency of eating legumes per week (-0.011, statistically insignificant) - decrease in the frequency of consuming juice per week (-0.055, statistically insignificant) - decrease in the probability of eating all types of vegetables at least twice per week (-0.12, statistically insignificant) - increase in the probability of eating fruits at least twice per week (0.01, statistically insignificant) - increase in the probability of eating legumes at least twice per week (0.03, statistically insignificant) - increase in the probability of consuming juice at least twice per week (0.01, statistically insignificant) When MMSA-level home prices increase by 10,000 for homeowners: - increase in the frequency of eating all types of vegetables per week (0.001, - decrease in the frequency of eating fruit per week (-0.005, statistically insignificant) - decrease in the frequency of eating legumes per week (-0.002, statistically insignificant) - decrease in the frequency of consuming juice per week ( -0.001, statistically insignificant) - decrease in the probability of eating all types of vegetables at least twice per week (-0.01, statistically insignificant) - decrease in the probability of eating fruit at least twice per week (-0.03, *P* < 0.05) - decrease in the probability of eating legumes at least twice per week (-0.02, *P* < 0.01) - decrease in the probability of consuming juice at least twice per week (-0.01, statistically insignificant)
**Ratcliffe (2015)** [40] **N:** 115 postcodes **Location:** United Kingdom **Timeframe:** 1991–2007	**Data:** Cross Section study using the BHPA between 1991–2007, contains General Health Questionnaire (12 questions aggregated to produce a 0–36 Likert scale value), house prices are matched using postal code data **Analysis:** Correlations and proxies of area quality	**Outcome:** Mental well-being assessed using the GHQ 12 question survey **Explanatory**: House price fluctuations **Mediating/Moderating:** Homeowners (outright owners); Homeowners (mortgaged); Renters in private renting; Renters in social renting **Confounding/Controlling:** Age; Marital status; Household consumption; Income; Macroeconomic shocks; New or old homeowners with mortgages	-The results for this study show that a 1% increase in local house prices, leads to an increase in the General Health Questionnaire by 0.005–0.006 units for both homeowners and renters (*p* < 0.05)
**Joshi (2016)** [41] **N:** individuals less than age 65 in BRFSS between 2005–2011 (exact number not given) **Location:** United States **Timeframe**: 2005–2011	**Data:** Longitudinal study on individual level data from the BRFSS, monthly county-level Zillow Home Value Index (ZHVI) as a proxy for local house prices **Analysis:** Exploiting a fixed-effects mode, regression coefficients	**Outcome:** Dependent variable, poor mental health days, from the response to the following question in the BRFSS: “Now thinking about your mental health, which includes stress, depression, and problems with emotions, how many days during the past 30 days was your mental health not good?” **Explanatory:** Housing prices using Zillow **Mediating/Moderating:** Predicted home ownership distribution **Confounding/Controlling:** Age; Gender; Education; Race/Ethnicity; Marital status; Unemployment rates; Country fixed effects	-For those with a predicted home ownership level at 25th percentile, a 1% decrease in house prices leads to a 0.0050 increase in poor mental health days - For those with a predicted home ownership level at 75th percentile, a 1% decrease in house prices leads to a 0.0029 increase in poor mental health days

### Risk of bias assessment

During the data extraction process, we conducted an assessment based on the Joanna Briggs Institute Critical Appraisal Tools [42]. Each study was classified according to its study design and rated using the appropriate tool designed for each study. However, despite varying methodological quality, no studies were excluded based on risk of bias assessment, as there were no clear sources of systematic bias with sufficient likelihood of challenging the conclusions of the source studies.

### Narrative synthesis

During the data extraction and risk of bias assessment phases, AG and LW recorded general notes on each of the studies. These notes, along with the extracted information, were used to construct a narrative synthesis of the evidence. This process was guided by Popay et al.’s [43] Guidance for Narrative Synthesis in Systematic Reviews. A narrative approach was selected to allow for an examination of the potential complexity inherent in the synthesis of findings across contexts, time periods, and populations to provide a nuanced discussion of what roles housing and rental markets might play in shaping health, with attention to both outcomes and potential mechanisms. Findings within study classes were reviewed to determine potential mediation and moderation. These explorations informed the development of a list of key points used to organize the presentation of our results. We then integrated and contextualized these findings with those from other relevant (though excluded) studies identified through our review process and from the texts of the included articles.

## Results

### Included studies

**Fig. 1:**
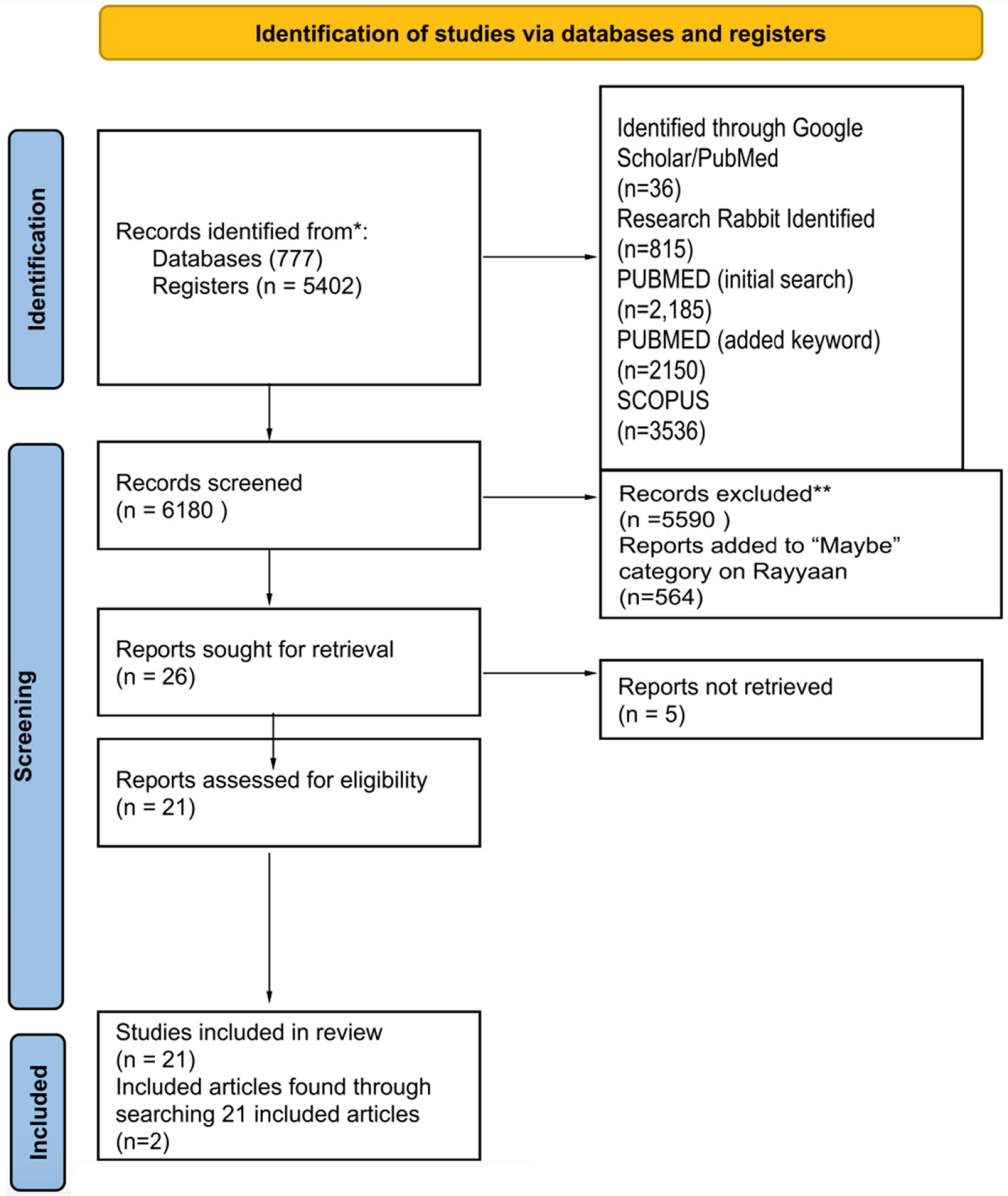
PRISMA systematic review flow diagram

Our keyword search returned 6,180 articles. Of these, 5,590 were removed based on review of the abstract and title as they were not directly related to our review topic (i.e., they did not measure changes in housing price and/or health outcomes). The remaining articles were reviewed based on their full-texts and a final list of 26 articles were considered for inclusion. However, five articles were not able to be retrieved (even after emailing the original authors), leaving us with 21 articles. The reference lists and bibliographies for these 21 included articles were then screened and two additional articles were thus included in our review resulting in a final sample of 23 articles. Figure 1 shows the flow diagram for included studies and these studies are listed in Appendix B.

### Dates and locations of studies

A full description of studies is included in Table 1. Studies were published from 2013–2022. Ten studies were from East Asia, eight from the United States, three in Europe, one from Australia, and one included nine countries (France, Japan, Netherlands, Spain, Switzerland, Sweden, United Kingdom, USA).

### Study design

Of the included studies, ten had a longitudinal study design, and thirteen studies were serial cross-sectional studies. Studies examined the effect of housing prices on health over time by repeatedly surveying a specific geographical area or population. One study included both qualitative and quantitative data collection.

### Outcome variable measurement

Most studies compared multiple outcomes. Seven studies focused on mental health as the outcome variable—utilizing various measures, including self-rated mental health, standardized scales for depression or anxiety, and receipt of pharmaceutical prescriptions [22, 32, 33, 35, 40, 41]. Nine studies analyzed the impact of housing prices on physical health—utilizing various measures of physical health, including objective assessments of physical health (e.g., body mass), self-rated physical health assessments, reports of specific health conditions (e.g., COVID-19), reported health behaviours (e.g., alcohol use, smoking), and mortality ( [24, 28, 29, 37, 44, 30, 36, 38, 39]). Seven studies included both physical and mental health measures as their outcome variable [19, 20, 23, 26, 27, 34, 45].

### Explanatory variable measurement

Housing prices were measured using many different types of data, including house price index, self-reported housing price (extracted from surveys), and average market price. Many studies used house price index as a measure of housing prices [19, 21, 22, 30, 38, 39, 41]. Zhang & Zhang [33] included self-reported housing price. Alternatively, many studies examined housing market prices using existing survey data [20, 23–29, 31, 32, 34–37, 40].

### Key findings

Studies included in our review highlighted a plurality of results when testing the relationships between housing prices and health. As shown in Tables 2 (physical health) and Table 3 (mental health), the included articles reported mixed findings across the outcomes explored. Given the heterogeneity of findings regarding the associations between housing prices and health outcomes, several authors examined potential moderators and mediators in attempt to understand the mechanisms at play. These included studies examine the role of wealth effects (by comparing effects on homeowners and renters), socioeconomic status (e.g., income level), and broader economic forces (e.g., area-level improvements). While keeping these pathways in mind, there are likely other alternative explanations beyond those explored. However, these appear to be the most dominant frameworks used to understand the effects in our included studies.

**Table 2:** Narrative overview of primary themes and findings, with citations for relevant articles (Physical Health)

	**Owners**			**Renters**	**All (non-disaggregated)**
	**Outright Owner**	**Mortgaged Owner**	**All Owners (Disaggregated)**		
Negative Effect	Wang & Liang (2021) [31]	Wang & Liang (2021) [31]	De & Segura-Escano (2021); Wong et al. (2020). Sung & Qiu. (2020) [30, 38, 39]	Atalay et al. (2017); Wang & Liang (2021); Sung & Qiu. (2020); De & Segura-Escano (2021) [27, 30, 38]	Chen et al. (2021); Arcaya et al. (2020); Yuan et al. (2020); Wei et al. (2021); Xu & Wang (2021) [20, 21, 26, 32, 36]
Positive Effect	Yue & Ponce (2021); Atalay et al. (2017); Wang & Liang (2021) [19, 27, 31]	Yue & Ponce (2021). Wang & Liang (2021) [19, 31]	Fichera & Gathergood (2016); Feng & Nie (2022); Hamoudi, & Dowd (2013); Ratcliffe (2015) [23, 34, 37, 40]	Yue & Ponce (2021); Ratcliffe (2015). Wang & Liang (2021); Sung & Qiu. (2020) [19, 31, 38, 40]	Kim et al. (2021); Daysal et al. (2021); Bao et al. (2022) [24, 28, 29]
Null Effects	Yue & Ponce (2021) [19]	Yue & Ponce (2021) Atalay et al. (2017) Wang & Liang (2021) [19, 27, 31]	Hamoudi, & Dowd (2013 Sung & Qiu. (2020) Wong et al. (2020) [37–39]	De & Segura-Escano (2021) Wang & Liang (2021) Hamoudi, & Dowd (2013) Sung & Qiu. (2020) Wong et al. (2020) [30, 31, 37–39]	**Chen et al. (2021**) Daysal et al. (2021). Wei et al. (2021) [20, 29, 32]

**Table 3:** Narrative overview of primary themes and findings, with citations for relevant articles (Mental Health)

	**Owners**			**Renters**	**All (non-disaggregated)**
	**Outright Owner**	**Mortgaged Owner**	**All Owners (Non-Disaggregated)**		
Positive Effect		Wang & Liang (2021) [31]	Chun et al. (2020); Zhang & Zhang (2019) [33, 35]	Yue & Ponce (2021); Hamoudi & Dowd (2014); [19, 25]	Chen et al. (2021) [20]
Negative Effect	Wang & Liang (2021) [31]		Hamoudi & Dowd (2014); Feng & NIe (2022); Joshi (2016); Sung & Qiu. (2020); Joshi (2016) [25, 34, 38, 41]	Atalay et al. (2017); Sung & Qiu. (2020); Joshi (2016); Wang & Liang (2021); [27, 31, 38, 41]	Lee et al. (2021); Yuan et al. (2020); Wei et al. (2021); Joshi (2016) [22, 26, 32, 41]
Null Effects	Yue & Ponce (2021) Atalay et al. (2017) [19, 27]	Yue & Ponce (2021) Atalay et al. (2017) [19, 27]	Fichera & Gathergood (2016) [23]	Chun (2020) [35]	Chen et al. (2021) [20]

#### Wealth effects

The first major pathway has been described as a “wealth effect” – which produces different effects for homeowners and renters [19, 20, 23, 25, 27, 33, 35, 37, 45]. For example, Hamoudi & Dowd [37] report that homeowners living in areas with steep price increases, perceive this as an increase in their overall wealth, resulting in positive health outcomes (not observed for renters). Similarly, Zhang & Zhang [33] show that increases in house prices has a positive effect on homeowner’s subjective well-being. De & Segura [30] specifically notes that price depreciation causes homeowners to experience feelings of a loss of wealth, leading to increases in alcohol consumption. Among studies that fail to show a wealth effect, Daysal et al. [29] shows that rising prices in Denmark do not impact households due to the buffering effects of government supports. Conversely, when examining the effects among renters, Wang & Liang [31] argue that rising housing prices have detrimental "strain" effect, which is also observed in several studies included in our review [25, 27, 38, 39, 45].

#### Income level

In addition to the wealth and strain effects illustrated through studies among homeowners and renters, many studies also examined the mediating effects of income [19, 20, 22, 28–30, 32, 33, 35, 38–40]. Several of these studies show that housing unaffordability constrains spending and that low-income individuals are particularly impacted [22, 24, 33, 38]. For example, Wong et al. [39] show that housing prices lead to reduced fruit consumption. However, results also show positive impacts for low-income homeowners – as exemplified by work showing that low-income homeowners are more sensitive to housing price gains [38, 40].

#### Broader economic forces

In considering both mechanisms described above, authors of included studies have also considered whether housing prices are merely an indicator of broader economic trends merits consideration. The most common strategy for accounting for this has been to include other indicators that might capture area level improvements. Indeed, most studies controlled for both individual characteristics or variables, such as age, gender, marital status, years of education, race/ethnicity, and employment status [20, 23–27, 29, 30, 32, 34, 35, 37, 39–41, 45], and a variety of economic factors, including individual income, country-level median income, and local area characteristics [19, 22–28, 30, 32, 36–40, 44, 45]. These factors are important to control for because rising housing prices can indicate a growing economy in which there are substantial improvements to neighbourhoods and communities [27, 29, 33, 38, 40]. As such, the observed improvements in health could simply arise from broader economic benefits (rather than being specifically attributable to housing prices) [31, 33]. However, generally speaking studies showed that there were independent effects of housing price or value, even after controlling for local area level improvements, and wider economic conditions [19, 27, 38].

### Strength of effects

Given heterogeneity in the direction of effects, the lack of standardization in the reporting of effect sizes from study to study, differences in the measurement of exposure and outcome variables, and variation in the inclusion of mediators, moderators, and confounders, we did not conduct a meta-analysis to describe the effect size of housing price on health. However, housing prices appear to exert influence on health and wellbeing with statistically significant effects across various health-related outcomes (See Table 1 for range of effect measures). The effects generally appear to be smaller when considering specific health conditions and greater when considering more subjective and more broad definitions of health (e.g., self-rated health). Of course, at a population-level, even relatively small effect sizes may pose a considerable challenge. For example, Xu & Wang [36] report that a 10% increase in housing prices is associated with a 6.5% increase in probability of reporting a chronic disease – a relatively small increase on a person-level, but when scaled could easily pose a considerable burden to the health system. In summary, further careful measurement and methodological refinement is needed to quantify the effects of housing prices on various health conditions. For any given health condition, this will require multiple well-designed studies across place and time. Such replication is particularly important given the observed sensitivity of findings to the inclusion of confounders, moderators, and mediators.

## Discussion

### Primary findings

While examining whether changes in housing prices are associated with changes in health, we recognized it is difficult to establish a directional and causal relationship between housing price and health. This is particularly true given that health may increase opportunities for home ownership and economic success [3, 46–49]. Nevertheless, given the wealth of literature highlighting housing affordability as a key determinant of health [7, 9, 10], it is reasonably anticipated that rising housing prices would be associated with worse health outcomes among individuals who do not own housing. However, based on analyses of the studies included in this review, the relationship between housing price and health is complex and nuanced, with a significant degree of heterogeneity across outcomes and populations.

First, this review illuminates that changing housing prices impacted different people differently, depending on for example, income level, gender and/or homeownership status [19, 22, 25]. The negative impact of housing price on health for renters and low-income individuals could be due to the existential angst from being excluded from home ownership, which is often considered an important indicator of social class and success [8]. However, this could also be due to the cost effect that is created from the rising house prices, subsequently raising low-income owners and renters’ cost of living [31]. Additionally, renting may be associated with lower neighbourhood tenure, especially when individuals are priced out of a neighbourhood [8]. As a result, they may experience deleterious health effects associated with loneliness, social isolation, lack of neighbourhood cohesion, and community disconnectedness [8]. Likewise, the positive effect observed among homeowners and high-income individuals may be explained by increases in psychological safety leading to changes in health behaviors, for example, knowing they have invested in a home that will support them or their heirs financially, people may be better able to focus on their well-being. As well, homeowners may be able to directly leverage the value of their home to gain access to additional capital and investment opportunities – which could support increased financial wellbeing [8].

The effects of housing on health can be conceptualized as arising from two sources. It appears that rising “cost effects” (the increased costs of houses and the costs passed on to tenants) are inversely correlated with health while “wealth effects” (the contributions of housing price to person wealth) contribute positively to health (for example, for homeowners and investors whose wealth increases due to the rising cost of housing). The balance of these effects differs depending on their unique impact on individuals – with lower income people and renters more strongly impacted by cost effects, and higher income people and homeowners more strongly impacted by wealth effects.

In considering these effects, we note that there is likely considerable geographic, temporal, and contextual variation in the health effects of rising housing prices. For example, rising housing prices may occur alongside neighbourhood improvements (or degradation) and economic booms (or recessions) [45], which themselves are associated with improvements (or deterioration) to health [8]. As such, the presence of these factors may obscure or interact with the gains to health. Similarly, variations across cultures and countries may change how individuals internalize the rising housing prices [50], causing them to experience greater or lesser distress in reaction to rising prices.

#### Limitations of included studies and directions for future research

Given these two primary factors, research highlights several opportunities for improving this literature. First, future studies should give more careful attention to how moderators and mediators are conceptualized. For example, “home-owners” are hardly a homogenous class of individuals: some own their homes outright and others are paying mortgages that offer varying levels of security (e.g., fixed vs. variable mortgages, 5-year vs. 30-year mortgages) [8]. Second, a broader range of effect moderators should be explored. For example, few studies specifically examined the health effects of rising home prices on vulnerable populations, including young adults and first time home buyers who may be especially disadvantaged by rapidly growing housing prices [50]. Similarly, isolating effects as arising from economic, legal, environmental, and social pathways can help identify strategies for mitigating health harms. For example, it may be important to understand whether changes to neighbourhood environments drive health harms as opposed to changes in personal financial status. Third, more within-person studies are needed to understand the potential mechanisms and situational factors that promote or mitigate the health effects of rising housing prices. Along with use of appropriate, theoretically informed moderators, isolating the within-person effects can help us better quantify the effects of interest to inform policies and prevention strategies. Fourth, longer follow-up times may allow for better understanding regarding the time-horizons of the effects explored. Indeed, it is possible that rising housing prices could have differential effects on the health of a population in the short versus long term. This is particularly important given the interaction between housing prices (which may act as a price signal for investments) and other economic factors with strong potential to increase health [8]. Fifth, the studies used a variety of measures for housing price and health outcomes – which varied in quality. For example, health outcomes were primarily measured using self-reported measures [19, 20, 23, 25–27, 30, 31, 33–35, 37–41] – which may be highly sensitive to bias due to the likelihood that individuals might report worse health when they are unhappy with economic factors. Improving measurement of outcomes can be done by leveraging administrative and other data sources. Sixth, it can be difficult to link area-level and individual-level factors, particularly in the context of limited cross-sectional studies or in longitudinal studies with only a few follow-up points. Likewise, many cohort-based studies have limited geographic coverage or insufficient temporal scope. As such, longer, larger, and wider studies are needed to fully ascertain the relationships under consideration.

#### Implications of findings

Although further research is required to overcome the limitations mentioned, existing evidence indicates that increases in housing prices may significantly influence health outcomes. Future studies should aim to exclude alternative explanations, examine the effects over longer periods, and establish consistent measurement methods to predict the impact of housing prices more accurately on health. The findings of those students will aid policymakers in creating strategies that address the health implications of rising housing prices. Policy makers should develop frameworks that respond to the impacts of rising housing prices on health. Such approaches could be facilitated through frameworks such as the WHO’s Health in All Policies (HiAP) policy, which advocates for the inclusion of health and social impacts among other criteria used throughout decision making processes [51]. Many studies in this review support this view and describe their work as having important implications for housing and health policy [19–21, 24, 26–29, 31, 34, 35, 37–39]. For example, Yuan et al. [26] notes the importance of directing government support and housing subsidies towards vulnerable groups – though these should be packaged with other policies [26]. Such supports can apparently buffer against the negative effects of rising housing prices by creating a saftey net that reduces the psychosocial and cognitive effects associated with economic changes in one's personal circumnstance. Arcaya et al. [21] also recommends governments investigate establishing more mental health facilities in areas where housing price fluctuations impact people's mental health but warns economic development that allows for greater investment in health infrastructure can also lead to increases in housing prices.

Of course, other types of interventions may also be warranted, including broader financial interventions (e.g., direct loans; [52], those which promote community, neighborhood, and social cohesion among residents [53], or those that aim to change how people value home ownership [26]. With respect to this final option, communities should consider whether renting may in fact be a desirable outcome for some individuals and therefore promote a culture in which individuals realize the variety of investment opportunities available to them rather than being overly-focused on a traditional model of investment [26, 32]. For example, Zhang & Zhang [33] writes that homeowners should be provided financial and economic knowledge to better manage wealth gains, however, this could be taken one step further and include the importance of educating people on the dangers on the commodification of housing, to prevent an over reliance on the importance of housing wealth gains.

#### Limitations of our review

In addition to the limitations specific to studies included in our review, our review itself also has several limitations. First, while we trained two reviewers to conduct article screening, assessed inter-rater reliability as greater than 80%, and adjudicated conflicts with the help of a third reviewer, it is possible some articles that could have been included were excluded due to the many different forms of outcome and explanatory variable measurement. Second, while we have searched multiple databases, used comprehensive key words for our search, and conducted manual searches of the reference lists, it is possible there were studies that we missed and failed to include in this study. That said, it is unlikely the exclusion of these articles would change our conclusion that the literature is currently mixed and that there is a need to flesh out the mechanisms and moderators that link housing prices to health. Third, we were not able to conduct a meta-analysis, and our numeric reports of the number of studies with each characteristic should not be treated as a meta-analysis. Rather, these findings and analyses of these studies should be interpreted as a descriptive analysis that highlights significant heterogeneity of findings and critical inconsistencies in the mechanisms, mediators, and moderators that give rise to these associations. Fourth, we did not exclude any studies based on article quality because we did not find there to be sufficient heterogeneity in the quality of the observational studies to merit exclusion according to variable inclusion, study design, or sampling method. In other words, we sought to avoid introducing bias by arbitrarily excluding articles – particularly given that the number of articles captured here was already relatively low (at least given the diversity of methods, measures, and populations captured). However, future reviews might consider more narrowed inclusion and exclusion criteria when a sufficient body of literature is available for a given outcome. For example, limiting analyses to only well-designed cohort studies might support a more careful selection of articles. Finally, we note that we included studies across a wide variety of health outcomes. While this was done to maximize inclusion (given the wide heterogeneity of measures used), we acknowledge that future research might be strengthened by studying specific pathways linking housing prices to specific health and social outcomes. Such detailed research is greatly needed so as to not only highlight the relevance of housing prices to health but identify strategies for mitigating potential harms of rising housing prices.

## Conclusion

Our review shows that there are complex relationships between housing prices and health – with studies arriving to mixed conclusions across a wide-variety of health outcomes and populations. Yet, there is insufficient evidence for a causal relationship, but it appears that if such a relationship exists it likely differs according to homeownership status, income-level, and as a factor of the broader economic and structural forces in play, including the level of economic supports provided by governments for low income individuals. Future research should explore these pathways, moderators, and confounders using long-term, geographically diverse, cohort studies that account for a broad diversity of causal or alternative mechanisms. Such future research will allow for a more nuanced understanding of health and health inequities related to rising housing prices.

## Supplementary Information

**Supplementary Material 1. **

## Data Availability

All data generated or analysed during this study are included in this published article and its supplementary information files.
