# Supplementary material for: The impact of housing prices on residents’ health: a systematic review
Source: BMC Public Health. 2024 Apr 1;24:931. doi: 10.1186/s12889-024-18360-w (PMC10983630; doi:10.1186/s12889-024-18360-w)
Supplement: Supplementary file 1 — Supplementary Material 1. [file 12889_2024_18360_MOESM1_ESM.pdf]

Appendix A

After identifying a few relevant articles, we added them to the Research Rabbit bibliography software [73], which we used to identify closely related articles through co-citation and co-authorship networks. From these identified articles, we then developed keyword lists related to the two primary concepts studied in this review, which were (1) health, and (2) housing price. Several closely related systematic reviews were also examined to ensure that our keywords were appropriately selected [74–78]. The final Boolean search string syntax was as follows: *("health" OR "well-being" OR "wellbeing" OR "stress" OR "financial strain" OR anxiety OR "depression" OR "psychological stress") AND ("housing price" OR "housing cost" OR "rent" OR "rental" OR "lease" OR "home sales" OR "home values" OR "mortgage" OR "housing market" OR "rental market" OR "housing appreciation" OR "repossession" OR "home ownership" OR "housing affordability" OR "housing wealth" OR "interest rates").*

After searching PubMed and SCOPUS using the keywords above, the abstracts of the articles were reviewed by two researchers to identify eligible articles using Rayyan. 6180 articles were screened for eligibility and a total breakdown of the number of articles identified from each database or register is provided in Figure 1, PRISMA Systematic Review Flow Diagram below. If the relevance of each article could not be determined from its abstract, the full manuscripts were reviewed. Eligible criteria were related to two main points; 1. original empirical research studies published in English prior to June 1, 2022 (when our search was conducted) and 2. articles must directly addressed our research question. Articles were not excluded based on geographic location or year.

APPENDIX B.

Included Articles, By Search Stage (N = 23)

Stage 1. Articles Identified from Initial Rudimentary Searches (N = 6)

1. Wang, H.-Q., & Liang, L.-Q. (2022). How Do Housing Prices Affect Residents' Health? New Evidence from China. *Frontiers in Public Health*, *9*. Scopus. https://doi.org/10.3389/fpubh.2021.816372

2. Yue, D., & Ponce, N. A. (2021). Booms and Busts in Housing Market and Health Outcomes for Older Americans. *Innovation in Aging*, *5*(2), igab012. https://doi.org/10.1093/geroni/igab012

3. Daysal, N. M., Lovenheim, M., Siersbæk, N., & Wasser, D. N. (2021). Home prices, fertility, and early-life health outcomes. *Journal of Public Economics*, *198*. Scopus. https://doi.org/10.1016/j.jpubeco.2021.104366

4. Feng, Y., & Nie, C. (2022). The effect of housing price on health: new evidence from China. *Applied Economics Letters*. Scopus. https://doi.org/10.1080/13504851.2022.2040726

5. Yuan, W., Gong, S., & Han, Y. (2020). How does rising housing price affect the health of middle-aged and elderly people? The complementary mediators of social status seeking and competitive saving motive. *PloS One*, *15*(12), e0243982. https://doi.org/10.1371/journal.pone.0243982

6. Xu, Y., & Wang, F. (2022). The health consequence of rising housing prices in China. *Journal of Economic Behavior & Organization*, *200*, 114–137. https://doi.org/10.1016/j.jebo.2022.05.011

Stage 2. Articles Identified Using Research Rabbit (N =7)

1. Hamoudi, A., & Dowd, J. B. (2013). Physical health effects of the housing boom: Quasi-experimental evidence from the health and retirement study. *American Journal of Public Health*, *103*(6), 1039–1045. https://doi.org/10.2105/AJPH.2012.301205

2. Zhang, C., & Zhang, F. (2019). Effects of housing wealth on subjective well-being in urban China. *Journal of Housing and the Built Environment*, *34*(4), 965–985. Scopus. https://doi.org/10.1007/s10901-019-09651-5

3. Wei, G., Zhu, H., Han, S., Chen, J., & Shi, L. (2021). Impact of house price growth on mental health: Evidence from China. *SSM - Population Health*, *13*, 100696. https://doi.org/10.1016/j.ssmph.2020.100696

4. Sung, J., & Qiu, Q. (2020). The Impact of Housing Prices on Health in the United States Before, During, and After the Great Recession. *Southern Economic Journal*, *86*(3), 910–940. Scopus. https://doi.org/10.1002/soej.12411

5. Atalay, K., Edwards, R., & Liu, B. Y. J. (2017). Effects of house prices on health: new evidence from Australia. *Social Science & Medicine (1982)*, *192*, 36–48. https://doi.org/10.1016/j.socscimed.2017.09.008

6. Chen, N., Shen, Y., Liang, H., & Guo, R. (2021). Housing and Adult Health: Evidence from Chinese General Social Survey (CGSS). *International Journal of Environmental Research and Public Health*, *18*(3). https://doi.org/10.3390/ijerph18030916

7. Chun, H. (2020). Do housing price changes affect mental health in South Korea? *Ethiopian Journal of Health Development*, *34*(3), 48–59.

Stage 3. Articles Identified from Scopus and Pubmed via Title, Abstract, and Full Text Search (N = 8)

1. Wong, E. S., Oddo, V. M., & Jones-Smith, J. C. (2020). Are Housing Prices Associated with Food Consumption? *International Journal of Environmental Research and Public Health*, *17*(11). https://doi.org/10.3390/ijerph17113882

2. Lee CY, Chen PH, & Lin YK. (2021). An Exploratory Study of the Association between Housing Price Trends and Antidepressant Use in Taiwan: A 10-Year Population-Based Study. *International Journal of Environmental Research and Public Health*, *18*(9). https://pubmed.ncbi.nlm.nih.gov/33946567/

3. Kim, I. (2021). Spatial distribution of neighborhood-level housing prices and its association with all-cause mortality in Seoul, Korea (2013-2018): A spatial panel data analysis. *SSM - Population Health*, *16*, 100963. https://doi.org/10.1016/j.ssmph.2021.100963

4. Hamoudi A & Dowd JB. (2014). Housing wealth, psychological well-being, and cognitive functioning of older Americans. *The Journals of Gerontology. Series B, Psychological Sciences and Social Sciences*, *69*(2), 253–262.

5. Fichera, E., & Gathergood, J. (2016). Do Wealth Shocks Affect Health? New Evidence from the Housing Boom. *Health Economics*, *25 Suppl 2*(Suppl Suppl 2), 57–69. https://doi.org/10.1002/hec.3431

6. De, P. K., & Segura-Escano, R. (2021). Drinking during downturn: new evidence from the housing market fluctuations in the United States during the Great Recession. *Economics and Human Biology*, *43*, 101070. https://doi.org/10.1016/j.ehb.2021.101070

7. Bao, W., Tao, R., Afzal, A., & Dördüncü, H. (2022). Real Estate Prices, Inflation, and Health Outcomes: Evidence from Developed Economies. *Frontiers in Public Health*, *10*, 851388. https://doi.org/10.3389/fpubh.2022.851388

8. Arcaya, M. C., Nidam, Y., Binet, A., Gibson, R., & Gavin, V. (2020). Rising home values and Covid-19 case rates in Massachusetts. *Social Science & Medicine (1982)*, *265*, 113290. https://doi.org/10.1016/j.socscimed.2020.113290

Stage 4. Articles Identified from Bibliographies of Other Included Articles (N = 2)

1. Joshi, N. K. (2016). Local house prices and mental health. *International Journal of Health Economics and Management*, *16*(1), 89–102. https://doi.org/10.1007/s10754-015-9177-x

2. Ratcliffe, A. (2015). Wealth Effects, Local Area Attributes, and Economic Prospects: On the Relationship between House Prices and Mental Wellbeing. *Review of Income and Wealth*, *61*(1), 75–92. https://doi.org/10.1111/roiw.12075
